# Antibacterial Effect of the In Vitro Interaction Between Clotrimazole and Chlorhexidine Against *Staphylococcus pseudintermedius* With Known Resistance Patterns Isolated From Canine Pyoderma

**DOI:** 10.1002/vms3.71162

**Published:** 2026-08-11

**Authors:** Brenda Gallegos‐Torres, Edgar Alfonseca‐Silva, Claudia Ledesma‐Carrasco, Itzcoatl Aquino‐Díaz

**Affiliations:** ^1^ Department of Physiology and Pharmacology Facultad de Medicina Veterinaria y Zootecnia Universidad Nacional Autónoma de México (UNAM) Mexico City Mexico; ^2^ Department of Microbiology and Immunology Facultad de Medicina Veterinaria y Zootecnia Universidad Nacional Autónoma de México (UNAM) Mexico City Mexico; ^3^ Holland Animal Health Jiutepec Morelos Mexico

**Keywords:** antimicrobial resistance, chlorhexidine, clotrimazole, drug interaction, FIC index, *Staphylococcus pseudintermedius*

## Abstract

**Background:**

*Staphylococcus pseudintermedius* is the main etiological agent of canine pyoderma and exhibits increasing multidrug resistance. Due to the reduced efficacy of antimicrobials, topical combinations of antimicrobial agents—such as chlorhexidine (CHX) and clotrimazole (CLT)—have gained attention as an alternative.

**Objective:**

To evaluate the in vitro antibacterial activity and pharmacological interaction of a 2:1 CHX–CLT combination against *S. pseudintermedius* isolates from canine pyoderma.

**Materials and Methods:**

Sixteen isolates of *S. pseudintermedius* were obtained from canine pyoderma and identified by PCR detection of the *nuc* gene. Methicillin resistance was assessed both phenotypically, by oxacillin disk diffusion, and genotypically, through *mecA* detection. Minimum inhibitory concentrations for CHX, CLT and their 2:1 combination were determined by broth microdilution using resazurin. The fractional inhibitory concentration index was calculated to assess drug interaction.

**Results:**

All isolates carried both *nuc* and *mecA* genes, while only 25% were phenotypically resistant to oxacillin. CHX exhibited MIC_50_ and MIC_90_ values of 0.781 and 1.562 µg/mL, respectively; CLT showed 6.25 and 12.5 µg/mL. In the 2:1 CHX–CLT combination, MICs were reduced up to 6‐fold, with MIC_50_ and MIC_90_ values of 0.39–0.781 µg/mL (CHX) and 0.195–0.39 µg/mL (CLT). The interaction was additive in 81.25% of isolates and indifferent in the remainder.

**Conclusions:**

The CHX–CLT combination demonstrated enhanced in vitro antibacterial activity with predominantly additive interactions against *S. pseudintermedius*. The results support the rational use of CHX–CLT topical formulations as an effective therapeutic alternative for canine pyoderma in the context of growing antimicrobial resistance.

## Graphical abstract

The 2:1 chlorhexidine–clotrimazole combination demonstrated enhanced antibacterial activity against canine *Staphylococcus pseudintermedius* isolates, including resistant strains. Reduced MIC values, predominantly additive interactions and absence of antagonism support its use as a promising topical strategy for canine pyoderma management.

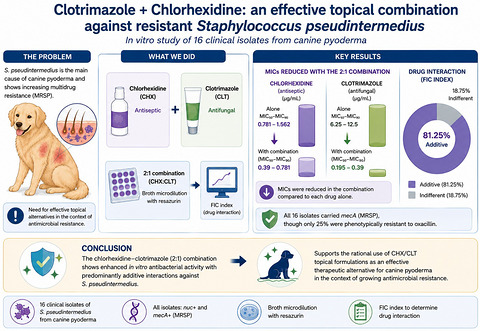

## 1 Introduction

Cutaneous infections are among the leading causes of antimicrobial prescription in dogs, with *Staphylococcus pseudintermedius* being the etiological agent responsible for most cases (Larsen et al. 2018; Lynch and Helbig 2021; Wang et al. 2025). This bacterium is a known resident of the skin and mucous membranes and a constituent of the microbiota of dogs. However, under certain conditions, like abnormalities of the skin condition, it can behave as an opportunistic pathogen causing infections of the skin, ears and other sites. In addition to its clinical significance in veterinary medicine, *S. pseudintermedius* is considered a potentially zoonotic agent capable of infecting humans—particularly immunocompromised individuals—posing a public health risk (Moses et al. 2023; Putriningsih et al. 2024).

Its ability to acquire resistance to various antimicrobials further exacerbates its clinical impact. In particular, the presence of the *mecA* gene encoding penicillin‐binding protein 2a (PBP2a), which has low affinity for β‐lactam antibiotics, confers resistance to methicillin and other β‐lactams. The *mecA* gene can be found in a mobile element of the bacterial chromosome known as the staphylococcal chromosomal cassette (SCCmec). Strains carrying this gene, known as methicillin‐resistant *S. pseudintermedius* (MRSP), often exhibit multidrug resistance, defined as resistance to at least one agent in three or more classes of antimicrobials (Aguayo‐Reyes et al. 2018; Putriningsih et al. 2024; Parasana et al. 2025).

Superficial pyoderma is likely to be the most frequent type of pyoderma in dogs and is characterized by epidermal invasion by bacteria. In most cases, *S. pseudintermedius* is the principal pathogen involved (Loeffler and Lloyd 2018). Topical treatment is the pillar for the management of bacterial skin infections in companion animals. Historically, it has often been regarded as a supplementary approach to systemic antimicrobial use. Nonetheless, recent guidelines and consensus statements emphasize the crucial role of topical antimicrobials in effectively addressing resistant bacterial skin infections (Santoro 2023).

Chlorhexidine (CHX) is a bisbiguanide compound with potent antibacterial and antifungal activity and is widely recommended for the topical treatment of superficial pyodermas (Clark et al. 2016; Santoro 2023). Its mechanism of action involves electrostatic interaction with negatively charged phospholipids and lipopolysaccharides in the bacterial cell wall, leading to membrane disruption, leakage of intracellular contents and cytoplasmic coagulation of proteins and nucleic acids (Abbood et al. 2023; Santoro 2023). Despite being considered a highly valuable option against resistant skin infections in dogs (Santoro 2023), recent studies have revealed potential cross‐resistance between CHX and other antimicrobials in adapted bacterial strains, possibly due to shared resistance mechanisms or selective pressure from extensive CHX use (Abbood et al. 2023).

To minimize resistance development in *S. pseudintermedius*, combining CHX with agents showing additive or synergistic effects has been suggested. Among commercial products for canine superficial infections are shampoos containing CHX and miconazole. Their MICs have been previously determined (MIC_90_ = 0.5 mg/L), showing additive or synergistic interactions (Clark et al. 2016)

In addition to their recognized antifungal properties, imidazoles such as miconazole, clotrimazole (CLT) and ketoconazole exhibit antibacterial activity against Gram‐positive bacteria, including antimicrobial‐resistant strains (Van de Vliet et al. 2024). Their antifungal action is due to inhibition of the cytochrome p450‐dependent enzyme lanosterol‐14­demethylase, essential for ergosterol synthesis. Their antibacterial activity might be based on the presence of 14α‐sterol‐demethylase­homologues in staphylococci. Recent studies also suggest that imidazoles exert antibacterial effects by inhibiting bacterial flavohemoglobins, which play key roles in nitrosative stress resistance and nitric oxide (NO) signalling (Nenoff et al. 2017).

Recently, the direct antibacterial activity of miconazole against *S. pseudintermedius* was evaluated by transmission electron microscopy, demonstrating that a concentration of 5 µg/mL alters bacterial membrane permeability and significantly reduces cell size (Voget et al. 2015; Santoro 2023).

Similarly, CLT has shown in vitro antibacterial activity against clinical *S. pseudintermedius* isolates. In fact, its performance was equal to or greater than that of miconazole, particularly against methicillin‐susceptible *Staphylococcus aureus* strains (Frosini and Bond 2017; Santoro 2023).

Given the need for new therapeutic alternatives, the CHX–CLT combination represents a promising option. Since both compounds have demonstrated individual antibacterial activity, their combination may enhance inhibitory effects and offer a novel therapeutic approach for dermal infections caused by *S. pseudintermedius*, including multidrug‐resistant strains.

The aim of this study was to: (1) determine the minimum inhibitory concentration (MIC) of CHX, CLT and their 2:1 combination against *S. pseudintermedius* strains isolated from canine dermal infections, regardless of their antimicrobial resistance profile; (2) compare the MIC values obtained for each individual compound with those from the combined formulation, in order to evaluate the pharmacological interaction between both agents; and (3) perform molecular identification of MRSP strains through PCR detection of the *mecA* gene and correlate the presence of this gene with the in vitro response to CHX, CLT and their combination.

## 2 Materials and Methods

### 2.1 Bacterial Isolates

A total of 16 clinical isolates of *S. pseudintermedius* obtained from dermal samples of dogs were provided by the Centro de Diagnóstico Veterinario Especializado (CEDIVETE). Preliminary identification of genus and species was carried out using conventional phenotypic and biochemical tests. Isolates were randomly selected from clinical cases of pyoderma documented between 2023 and 2024. Prior to inclusion in the study, all strains were tested for antimicrobial susceptibility by the agar diffusion method. Upon receipt, the strains were stored at −20°C in cryopreservation medium containing 10% skim milk and 10% glycerol. Colonies from the original culture media were suspended in sterile skim milk–glycerol solution, aliquoted (200 µL) into sterile cryovials and frozen until use.

### 2.2 Test Antibacterial Solutions

Three test formulations were evaluated: (1) 2% CHX, (2) 1% CLT and (3) a 2:1 mixture of CHX 2%: CLT 1%. In addition, a control solution (vehicle only, no active ingredients) was included. All solutions were donated by Holland Animal Health and produced according to the internal quality standards of the manufacturer.

### 2.3 Minimum Inhibitory Concentration Determination

MICs were determined using the broth microdilution method according to the Clinical and Laboratory Standards Institute (CLSI) guidelines, with modifications that included 0.02% sodium resazurin (FR08651, BIOSYNTH) as a redox‐based cell viability indicator (Elshikh et al. 2016). Prior to testing, strains were subcultured on tryptic soy agar (TSA; BD Bioxon) and incubated at 37°C for 16–24 h.

The test was performed in 96‐well flat‐bottom microtitre plates (Corning Costar). Initially, two serial 10‐fold and subsequently 2‐fold dilutions of the test solutions were prepared in nutrient broth. Final concentrations ranged from 0.048 to 20,000 µg/mL for CHX, from 0.024 to 10,000 µg/mL for CLT and from 0.012 to 10,000 µg/mL for the CHX–CLT mixture. Bacterial inoculum was adjusted to the McFarland turbidity standard 0.5 (≈1 × 10^8^ CFU/mL). The wells where the 10‐fold dilutions were made (1:10 and 1:100) received 180 µL of nutrient broth (MCD LAB 7342), and the wells where the rest of the 2‐fold dilutions were prepared received 100 µL. For the 1:1 dilution wells, 120 µL of each test solution was added. Subsequently, 20 µL of the 1:1 dilution was transferred to the well with 180 µL of medium to obtain the 1:10 dilution and homogenized; subsequently, 20 µL of this mixture was transferred to the next well with medium to prepare the 1:100 dilution. Subsequently, for double dilutions, successive transfers of 100 µL were made between the wells, until the dilution series was completed, discarding 100 µL from the last well. Finally, 100 µL of bacterial inoculum was added to each well.

Two control wells were included in each assay: (1) positive control (inoculum without treatment) and (2) excipient control (inoculum + solution excipient). For quality control purposes, one reference strain (*S. aureus* American Type Culture Collection [ATCC] 29213) was included. Plates were incubated at 37°C for 24 h, followed by addition of 30 µL of 0.02% resazurin (filtered with 0.45 µm syringe filter, Corning). Plates were incubated again at 37°C for 24 h. Colour change from blue to pink indicated bacterial growth.

To confirm MIC values, 5 µL from colour‐shifted wells was plated on TSA in duplicate and incubated at 37°C for 24 h. Visual assessment of bacterial colony growth was used to determine MIC endpoints. MIC was recorded as the lowest concentration of the test solution that completely inhibited colony formation, without considering individual colonies. Two independent experiments were conducted, each with two internal replicates.

### 2.4 Fractional Inhibitory Concentration Index

The pharmacological interaction between CHX and CLT was assessed by calculating the fractional inhibitory concentration index (FICi), using the formula:

$$\Sigma \text{FIC}\;=\;{\text{FIC}}_{A}\;+\;{\text{FIC}}_{B}\;=\;\left({\text{MIC}}_{\mathrm{AB}}/{\text{MIC}}_{A}\right)\;+\;\left({\text{MIC}}_{\mathrm{AB}}/{\text{MIC}}_{B}\right),$$

where MIC_A_ and MIC_B_ are the individual MICs of CHX and CLT, and MIC_AB_ is the MIC of the combination.

Results were interpreted using EUCAST criteria: ΣFIC ≤ 0.5 = synergy; > 0.5–1 = additive; > 1–< 2 = indifferent; and ≥ 2 = antagonism (EUCAST 2000; Clark et al. 2016).

### 2.5 Genotypic and Phenotypic Identification of Methicillin‐Resistant *S. pseudintermedius*

Molecular species identification was confirmed by PCR targeting the *nuc* gene, which encodes a thermonuclease specific to *S. pseudintermedius*. Methicillin resistance was assessed genotypically by detection of the *mecA* gene, and phenotypically by disc diffusion assay using 1 µg oxacillin discs (Oxoid) on Mueller–Hinton agar (BD Bioxon), according to CLSI guidelines.

A multiplex PCR was designed to detect *nuc* and *mecA* simultaneously. Primers were designed using NCBI Primer‐BLAST and synthesized by the UNAM Institute of Biotechnology (Table 1). PCR was performed using MyTaq 2× Mix (BIOLINE). DNA template was obtained by suspending a single colony in 10 µL of nuclease‐free water.

**TABLE 1:** Primer sequences used for multiplex PCR amplification of *nuc* and *mecA* genes from *S. pseudintermedius*.

Target gene	Amplicon size (bp)	Primer (5′–3′) sequence
*nuc*	376	Forward: ACG GAT GTG AGA GGC GAA TC
		Reverse: GTG CTT CCT TTT GGG CTT GT
*mecA*	141	Forward: CGA GAC TGT TGC AGA AAC GC
		Reverse: CGC TTC ACT TGT TCT ACC ACG

Abbreviation: bp, base pairs.

The PCR amplification protocol was as follows: initial denaturation at 95°C for 2 min; 30 cycles of denaturation at 95°C for 45 s, annealing at 64°C for 30 s and extension at 72°C for 45 s; followed by a final extension step at 72°C for 2 min.

Amplified PCR products were separated on 1.8% agarose gels stained with Diamond (PROMEGA), electrophoresed at 50 V for 10 min and then at 70 V for 30 min, and visualized using a gel documentation system (Axygen). As positive control, we used a laboratory strain of MRSP. A *S. aureus* strain was included as negative control.

### 2.6 Data Analysis

FIC indices were calculated using Microsoft Excel based on MIC values from individual and combined treatments.

## 3 Results

### 3.1 Antimicrobial Susceptibility Profile and Phenotypic Classification of Isolates

Phenotypic susceptibility data obtained by the agar diffusion method were compiled for 16 clinical isolates of *S. pseudintermedius* against 13 antimicrobials representing seven different families. Each isolate was classified as either susceptible or resistant according to standard interpretive criteria. The full results are presented in Table 2.

**TABLE 2:** Antimicrobial susceptibility profile of 16 clinical isolates of *S. pseudintermedius*.

Antimicrobial family	Antimicrobial agent	Resistant isolates (*n*)	Susceptible isolates (*n*)
β‐lactams	Oxacillin	4	12
	Amoxicillin–clavulanic acid	0	16
First‐generation cephalosporins	Cephalexin	3	13
	Cephalothin	0	14
Fluoroquinolones	Ciprofloxacin	5	8
	Enrofloxacin	4	12
Amphenicols	Florfenicol	0	9
Aminoglycosides	Gentamicin	6	10
	Neomycin	11	3
	Amikacin	1	2
Sulphonamides	Sulfamethoxazole–trimethoprim	3	2
Lincosamides	Clindamycin	2	2

High susceptibility was observed for amoxicillin–clavulanic acid, with all 16 isolates reported as susceptible. In contrast, neomycin exhibited the highest resistance rate, with 11 of the 16 isolates classified as resistant. Gentamicin followed with six resistant isolates.

Regarding oxacillin—the reference antimicrobial for phenotypic detection of methicillin resistance—four isolates were resistant, while 12 were susceptible. Resistance to cephalexin was observed in three isolates. Among the Fluoroquinolones, five isolates were resistant to ciprofloxacin and four to enrofloxacin. Three isolates showed resistance to sulfamethoxazole—trimethoprim. Resistance was also detected in two isolates to clindamycin and in one isolate for amikacin. No resistance was observed for cephalothin or florfenicol.

In order to comprehensively interpret the observed resistance patterns, the 16 *S. pseudintermedius* isolates were classified according to two main criteria: (1) phenotypic resistance to oxacillin, used as an indicator of methicillin resistance and (2) the number of antimicrobial families to which resistance was detected. This classification allowed for the grouping of isolates into four phenotypic categories, as shown in Table 3.

**TABLE 3:** Classification of *S. pseudintermedius* isolates according to oxacillin resistance and the number of antimicrobial families with phenotypic resistance.

Isolate	Oxacillin	No. of resistant families	Resistant families	Assigned group
**1**	R	4	β‐lactams, Fluoroquinolones, Aminoglycosides, Lincosamides	MRSP
**2**	S	0	—	Susceptible
**3**	S	1	Aminoglycosides	Specific resistance
**4**	S	0	—	Susceptible
**5**	S	2	Aminoglycosides, Sulphonamides	DR‐MSSP
**6**	S	0	—	Susceptible
**7**	R	3	β‐lactams, Fluoroquinolones, Aminoglycosides	MRSP
**8**	S	0	—	Susceptible
**9**	S	1	Aminoglycosides	Specific resistance
**10**	S	1	Aminoglycosides	Specific resistance
**11**	S	2	β‐lactams, Aminoglycosides	DR‐MSSP
**12**	R	4	β‐lactams, Fluoroquinolones, Aminoglycosides, Sulphonamides	MRSP
**13**	S	2	Fluoroquinolones, Aminoglycosides	DR‐MSSP
**14**	S	2	Fluoroquinolones, Aminoglycosides	DR‐MSSP
**15**	S	1	Aminoglycosides	Specific resistance
**16**	R	5	β‐lactams, Fluoroquinolones, Aminoglycosides, Sulphonamides, Lincosamides	MRSP

Abbreviations: DR‐MSSP, methicillin‐susceptible *S. pseudintermedius* resistant to two antimicrobial families; MRSP, methicillin‐resistant *S. pseudintermedius*; R, resistant; S, susceptible.

Four isolates (25%) were classified as MRSP based on resistance to oxacillin following CLSI guidelines. Another four isolates (25%) were classified as methicillin‐susceptible but resistant to two antimicrobial families (DR‐MSSP); these isolates were susceptible to oxacillin but exhibited resistance to two other antimicrobial families. Four additional isolates (25%) were grouped as specifically resistant strains, showing resistance to only one antimicrobial family other than oxacillin. Finally, the remaining four isolates (25%) showed no resistance to any of the tested agents and were classified as fully susceptible.

### 3.2 Minimum Inhibitory Concentration Determination

The MIC values for 2% CHX, tested against 16 clinical *S. pseudintermedius* isolates, ranged from 0.781 to 1.562 µg/mL (Table 4).

**TABLE 4:** Minimum inhibitory concentrations of chlorhexidine, clotrimazole and their 2:1 combination against 16 *S. pseudintermedius* isolates from canine pyoderma cases.

Drug	Group MIC 𝜇g/mL	0.195	0.390	0.781	1.562	3.125	6.25	12.5	25	MIC_50_	MIC_90_
Chlorhexidine 2%	Susceptible			4						0.781	0.781
	Specific resistance			4						0.781	0.781
	DR‐MSSP			2	2					0.781	1.562
	MRSP			3	1					0.781	1.562
	**Total *n* = 16**			**13**	**3**					**0.781**	**1.562**
Clotrimazole 1%	Susceptible						2	2		6.25	12.5
	Specific resistance					1	2	1		6.25	12.5
	DR‐MSSP						3	1		6.25	12.5
	MRSP						2	2		6.25	12.5
	**Total *n* = 16**					**1**	**9**	**6**		**6.25**	**12.5**
2:1 Chlorhexidine/clotrimazole chlorhexidine MIC	Susceptible		4							0.39	0.39
	Specific resistance		4							0.39	0.39
	DR‐MSSP		2	2						0.39	0.781
	MRSP			4						0.781	0.781
	**Total *n* = 16**		**10**	**6**						**0.39**	**0.781**
2:1 Chlorhexidine/clotrimazole clotrimazole MIC	Susceptible	4								0.195	0.195
	Specific resistance	4								0.195	0.195
	DR‐MSSP	2	2							0.195	0.39
	MRSP		4							0.39	0.39
	**Total *n* = 16**	**10**	**6**							**0.195**	**0.39**

Abbreviation: DR‐MSSP, methicillin‐susceptible *S. pseudintermedius* resistant to two antimicrobial families.

The MIC_50_ was 0.781 µg/mL and the MIC_90_ was 1.562 µg/mL. When analysing the bacterial groups, the susceptible isolates (*n* = 4) and those with specific resistance (*n* = 4) had a MIC_90_ of 0.781 µg/mL, while the DR‐MSSP (*n* = 4) and MRSP (*n* = 4) groups showed a higher MIC_90_ of 1.562 µg/mL. For 1% CLT, MIC values ranged from 3.125 to 12.5 µg/mL. The overall MIC_50_ was 6.25 µg/mL and the MIC_90_ was 12.5 µg/mL. These values were consistent across all phenotypic groups. The CHX–CLT combination showed consistently lower MIC values than either compound alone. For CHX within the combination, MIC values ranged from 0.39 to 0.781 µg/mL. The overall MIC_50_ and MIC_90_ were 0.39 and 0.781 µg/mL, respectively. Susceptible and specifically resistant isolates exhibited MIC_50_ and MIC_90_ values of 0.39 µg/mL; DR‐MSSP isolates had a MIC_50_ of 0.39 µg/mL and a MIC_90_ of 0.781 µg/mL; while MRSP isolates presented both values at 0.781 µg/mL. For CLT in the combination, MICs ranged from 0.195 to 0.39 µg/mL. The MIC_50_ and MIC_90_ were 0.195 and 0.39 µg/mL, respectively. Susceptible and specifically resistant isolates showed identical MIC_50_ and MIC_90_ values (0.195 µg/mL); DR‐MSSP isolates had a MIC_50_ of 0.195 µg/mL and a MIC_90_ of 0.39 µg/mL, and both values for MRSP isolates were 0.39 µg/mL. The quality control strain exhibited MIC values equal to or within one dilution of the test ranges.

### 3.3 Fractional Inhibitory Concentration Index

The FICi was evaluated for the 2:1 CHX–CLT combination in all 16 clinical isolates of *S. pseudintermedius*. Among the tested isolates, the interaction between both compounds was predominantly additive: 13 of the 16 strains (81.25%) showed an additive effect, while the remaining three strains (18.75%) exhibited an indifferent interaction. No antagonistic effects were observed in any of the isolates.

When results were analysed by phenotypic group, isolates classified as susceptible, specifically resistant and DR‐MSSP all displayed a uniform additive pattern (100%). In contrast, the MRSP group showed greater variability: one isolate (25%) demonstrated an additive interaction, while the other three (75%) showed an indifferent effect (Table 5).

**TABLE 5:** Interaction of the 2:1 chlorhexidine–clotrimazole combination determined by FIC index in 16 *S. pseudintermedius* isolates from canine pyoderma cases.

Treatment	Group	Synergy	Additivity	Indifference	Antagonism
2:1 Chlorhexidine/clotrimazole	Susceptible (*n* = 4)	0	4	0	0
	Specific resistance (*n* = 4)	0	4	0	0
	DR‐MSSP (*n* = 4)	0	4	0	0
	MRSP (*n* = 4)	0	1	3	0
	**Total (*n* = 16)**	**0**	**13**	**3**	**0**

Abbreviation: DR‐MSSP, methicillin‐susceptible *S. pseudintermedius* resistant to two antimicrobial families.

### 3.4 Genetic Identification of *S. pseudintermedius* and Detection of the Methicillin Resistance Gene

All analysed isolates tested positive for both the *nuc* and *mecA* genes, confirming their molecular identification as *S. pseudintermedius* and the presence of the genetic determinant for methicillin resistance, respectively.

## 4 Discussion

This study evaluated in vitro activity of CHX, CLT and their combination against *S. pseudintermedius* strains isolated from skin lesions in dogs, determining their MICs and analysing the type of pharmacological interaction between the two compounds. In addition, phenotypic susceptibility to 13 antimicrobials was assessed, and molecular identification of *mecA*‐positive strains was performed.

The bacterial population analysed in this study included both susceptible and resistant strains. A total of 75% of the *S. pseudintermedius* strains were classified as resistant to the antimicrobials tested; 25% exhibited resistance to a single class (Aminoglycosides); another 25% were classified as DR‐MSSP, showing resistance to Aminoglycosides and at least one additional class (β‐lactams, Sulphonamides or Fluoroquinolones), consistent with previously reported findings (Morais et al. 2023). Lastly, 25% were MRSP strains, exhibiting resistance to three to five different antimicrobial classes, a pattern also described in other studies (Usui et al. 2025).

Methicillin resistance is associated with the *mecA* gene, which confers resistance to all β‐lactam antibiotics. Moreover, MRSP strains frequently display cross‐resistance to other antimicrobial families such as Lincosamides, Fluoroquinolones, Aminoglycosides, Macrolides, Sulphonamides, Tetracyclines and Chloramphenicol (Morais et al. 2023; Pirolo et al. 2024). In this study, 25 % of the isolates were phenotypically resistant to oxacillin, a finding consistent with previous reports (Kalaba et al. 2020; Feßler et al. 2022; Morais et al. 2023), though lower than the prevalence reported in countries such as China (48.6%–55.8%), Japan (37%) and Italy (41%) (Iyori et al. 2021; Nocera et al. 2021; Y. Liu et al. 2024; Wang et al. 2025). However, the prevalence observed here was higher than those reported in Germany (12.2%), Finland (14%) and France (16.9%) (Grönthal et al. 2017; Haenni et al. 2020).

An unexpected finding was the complete (100%) susceptibility to amoxicillin–clavulanic acid, which contrasts with other studies that have reported high resistance rates (Kalaba et al. 2020; Iyori et al. 2021; Nocera et al. 2021). This discrepancy may be related to the inhibitory effect of clavulanic acid on β‐lactamases, as well as regional differences in the epidemiology of circulating strains in Mexico. Although a proportion of the isolates were classified as MRSP and are therefore considered resistant to β‐lactams, resistance to amoxicillin–clavulanic acid depends not only on the presence of PBP2a but also on the type and level of β‐lactamase expression (Ba et al. 2023). For cephalexin, resistance was low (18.75%), below the levels reported in the literature, which can reach up to 50% (Iyori et al. 2021). Aminoglycosides, especially neomycin, showed high resistance rates, with 37.5% for gentamicin and 78.57% for neomycin, values consistent with previous studies (Kalaba et al. 2020; Iyori et al. 2021). Fluoroquinolone resistance was 38.4% for ciprofloxacin and 25% for enrofloxacin, within the expected range according to prior reports (Nocera et al. 2021; Feßler et al. 2022; Morais et al. 2023). Sulphonamide resistance was 60%, significantly higher than the 30.3%–31.5% reported elsewhere (Iyori et al. 2021; Morais et al. 2023). Clindamycin resistance reached 50%, also higher than rates described in other studies, which range between 16.7% and 36.8% (Iyori et al. 2021; Morais et al. 2023). A positive finding was the complete absence of florfenicol resistance, consistent with a report from Japan (Usui et al. 2025), though contrasting with data from China, where resistance increased from 9.14% in 2018 to 20% in 2022 (Wang et al. 2025).

Although some strains were phenotypically susceptible, all tested strains carried the *mecA* gene, indicating their potential to express methicillin resistance. These findings align with the existing literature, which suggests that therapeutic options for MRSP infections in companion animals are limited, highlighting the need for susceptibility‐based strategies and rational antimicrobial use (Usui et al. 2025).

MIC values obtained for CHX, CLT and their combination revealed significant activity against all *S. pseudintermedius* strains, including MRSP. For 2% CHX, MIC_50_ and MIC_90_ were 0.781 and 1.562 µg/mL, respectively, differing by just one 2‐fold dilution, indicating a homogeneous susceptibility pattern among the tested strains. Even the least susceptible strains required concentrations far below those used in commercial products (2% = 20,000 µg/mL). These results agree with previous findings, which reported bactericidal concentrations of CHX ranging from 0.6247 to 7 µg/mL (Santoro 2023) and MIC values between 0.00003% and 0.0025% (equivalent to 0.3–25 µg/mL) (Feßler et al. 2022). A MIC_90_ of 2 mg/L (2 µg/mL) has also been documented (Clark et al. 2016), which is comparable to the values observed in this study and supports the efficacy of CHX even against resistant clinical isolates.

For 1% CLT, MIC_50_ and MIC_90_ were 6.25 and 12.5 µg/mL, respectively—values that are higher than those reported in previous studies, which found MIC_90_ values of 1 mg/L (1 µg/mL) in both susceptible and MRSP strains (Frosini and Bond 2017; Santoro 2023). This discrepancy could be related to methodological differences or regional strain variability. Nevertheless, even the highest MIC observed remained well below the concentration used in topical CLT formulations (1% = 10,000 µg/mL), supporting potential clinical efficacy against both susceptible and resistant strains. The efficacy of miconazole/CHX combinations and the individual activity of CLT against *S. pseudintermedius* have also been documented. A MIC_90_ of 2 µg/mL has been reported for miconazole against *S. pseudintermedius* (Clark et al. 2016; Santoro 2023). Although CLT showed a higher MIC_90_ (12.5 µg/mL) in this study, some reports suggest it may be equally or even more effective than miconazole, particularly in methicillin‐susceptible strains (Frosini and Bond 2017).

The 2:1 CHX–CLT combination demonstrated a clear potentiating effect, with MIC_50_ values reduced to 0.39 µg/mL (CHX) and 0.195 µg/mL (CLT), and MIC_90_ values to 0.781 and 0.39 µg/mL, respectively. This represents a 2‐ to 6‐fold reduction compared to the individual compounds, which is clinically significant, as lower concentrations were effective even against MRSP isolates. No isolate showed increased MICs when exposed to the combination. These findings are consistent with those reported for CHX/miconazole, where 75% of isolates exhibited MICs of 0.25–0.5 mg/L and up to 3‐fold reductions in CHX MICs (Clark et al. 2016). The activity of the CHX–CLT combination in this study was comparable in terms of MIC reduction.

According to the FICi, an additive effect was observed in 81.25% of the isolates, while the remaining 18.75% exhibited indifference and no antagonistic interactions were detected. Regarding previously published information on the interaction between imidazole derivatives and CHX, specifically the combination of miconazole–CHX, different outcomes have been reported. One study observed both synergistic and additive effects (Clark et al. 2015), whereas another reported only additive effects (Clark et al. 2016). The authors noted that these discrepancies may reflect some variability in drug activity and in MIC determinations, even when standardized protocols are followed (Clark et al. 2016). Nevertheless, regardless of whether additivity or synergy is observed, combinations of imidazoles with CHX consistently result in lower MICs compared to the drugs alone, making them a rational option for the treatment of canine superficial pyoderma (Clark et al. 2016). Although MRSP strains predominantly showed indifference, the MICs of the combination remained equal to or lower than the individual values, suggesting a therapeutic benefit. This could be attributed to complementary mechanisms of action: CHX disrupts the bacterial cytoplasmic membrane, while CLT might interfere with the synthesis of structural membrane components, as happens in fungi, due to the possible existence of 14α‐sterol‐demethylase‐homologues in bacteria (Nenoff et al. 2017). However, the precise mechanism of interaction remains to be elucidated.

The 2:1 ratio used in this study, consistent with some commercially available formulations, ensures therapeutic concentrations are achieved at the site of application, including in strains with decreased susceptibility or multidrug resistance. Collectively, these findings support the rational use of topical formulations combining CHX and CLT as an effective therapeutic alternative for the treatment of *S. pseudintermedius*‐associated pyodermas, including those caused by multidrug‐resistant strains such as MRSP. The observed reduction in MIC values for each active compound, the absence of antagonistic interactions and the predominance of additive effects reinforce their potential role as a first‐line treatment in the context of increasing antimicrobial resistance.

In the present study, all analysed isolates tested positive for both the *nuc* gene, specific to *S. pseudintermedius*, and the *mecA* gene, which confers resistance to methicillin. These results confirm the genotypic identity of the isolates as *S. pseudintermedius* and reveal a high prevalence of *mecA*, a molecular marker associated with resistance to β‐lactam antimicrobials.

Several studies have reported variable frequencies of *mecA*‐carrying *S. pseudintermedius* strains. For example, a prevalence of approximately 50% has been documented in Japan (Usui et al. 2025), while in South Africa, a frequency of 85.9% has been reported (Prior et al. 2022). In contrast, a 100% prevalence was observed in this study, which could be attributed to the origin of the samples, as they were collected from a diagnostic laboratory that receives clinical submissions from veterinary clinics and hospitals, where chronic conditions and prior antimicrobial exposure are common. This is consistent with previous reports indicating that dogs with a history of invasive procedures or chronic conditions, such as pyoderma, are at increased risk of harbouring methicillin‐resistant strains, likely due to greater hospital exposure and repeated antimicrobial treatments (Wang et al. 2025).

Despite universal detection of the *mecA* gene, only four isolates were classified as oxacillin‐resistant based on phenotypic testing, indicating a discrepancy between molecular detection and phenotypic resistance expression. This finding is not exclusive to the present study, as similar observations have been documented in previous reports. A *mecA*‐positive *S. pseudintermedius* strain that remained phenotypically susceptible to oxacillin has been described (Usui et al. 2025), reflecting a phenomenon also widely reported in *S. aureus* under the classification of oxacillin‐susceptible methicillin‐resistant *S. aureus* (OS‐MRSA). Consistent findings have also been noted in China, where a 4.5% prevalence of OS‐MRSA strains was identified. These strains carried the *mecA* gene but initially lacked phenotypic resistance, which could be induced following antimicrobial exposure (J. Liu et al. 2021). Possible explanations for this discrepancy include regulatory mechanisms affecting *mecA* expression or the influence of surrounding genetic elements. In particular, the frequent presence of the SCCmec Type IV element in OS‐MRSA—lacking multiple resistance genes—could explain why, although clinical MRSA strains often display a multidrug‐resistant phenotype, the OS‐MRSA strains are less likely to do so (J. Liu et al. 2021). Even so, the presence of OS‐MRSA strains presents important therapeutic challenges, as they may exhibit heteroresistance to oxacillin, increasing the risk of treatment failure if misclassified as susceptible. This underscores the need for both genotypic and phenotypic testing to fully characterize the resistance profile. Moreover, the presence of the *mecA* gene in strains that do not yet express phenotypic resistance may represent a latent risk for resistance development under antimicrobial pressure. Inappropriate or prolonged exposure to β‐lactams could induce *mecA* expression, favouring the selection of resistant subpopulations. This risk has been demonstrated in experimental settings, where all tested OS‐MRSA strains developed high‐level oxacillin resistance following antimicrobial exposure—highlighting the potential for phenotypic conversion under selective pressure (J. Liu et al. 2021). In this context, the rational use of antimicrobials becomes essential not only to ensure therapeutic efficacy, but also to prevent the emergence and spread of multidrug‐resistant strains.

Overall, these findings highlight the high prevalence of the *mecA* gene among clinical *S. pseudintermedius* isolates and reinforce the need to assess both its presence and phenotypic expression. This dual evaluation is essential to prevent therapeutic failures and to strengthen the epidemiological surveillance of MRSP.

To obtain more comprehensive insights in future studies, it is advisable to expand the geographical sampling area and increase the number of isolates analysed. Moreover, standardizing the antimicrobial susceptibility testing across all strains and incorporating molecular analyses to evaluate *mecA* gene expression would contribute to a more thorough understanding of resistance mechanisms and treatment potential.

## 5 Conclusions

The results of this study suggest that the CHX–CLT combination exhibits antimicrobial activity with predominantly additive interactions and no antagonism against clinical *S. pseudintermedius* isolates obtained from canine pyoderma cases. The universal detection of *mecA* underscores the need for integrative resistance profiling through both molecular and phenotypic methods. The observed genotypic–phenotypic mismatch supports the importance of rational antimicrobial use to prevent the emergence and spread of resistant strains. In this context, the evaluated 2:1 CHX–CLT formulation represents a promising topical alternative for treating *S. pseudintermedius*‐associated pyodermas, even in the presence of resistance to one or multiple antimicrobial classes.

## Author Contributions

**Brenda Gallegos‐Torres**: investigation, data curation, formal analysis, writing – original draft. **Edgar Alfonseca‐Silva**: methodology, project administration, writing – review and editing. **Claudia Ledesma‐Carrasco**: conceptualization, supervision, writing – review and editing. **Itzcoatl Aquino‐Díaz**: methodology, formal analysis, project administration, writing – review and editing. All authors have read and approved the final manuscript.

## Funding

This research was funded by the Universidad Nacional Autónoma de México (UNAM; www.unam.mx) through the PAPIIT Project IA208826. The funder had no role in the study design, data collection and analysis, decision to publish or preparation of the manuscript.

## Ethics Statement

The authors confirm that the ethical policies of the journal, as noted on the journal's author guidelines page, have been adhered to. No ethical approval was required as there were no animal subjects.

## Conflicts of Interest

The authors declare no conflicts of interest.

## Data Availability

The data that support the findings of this study are available from the corresponding author upon reasonable request.
